# Preparation of Poly-1-butene Nanofiber Mat and Its Application as Shutdown Layer of Next Generation Lithium Ion Battery

**DOI:** 10.3390/polym12102267

**Published:** 2020-10-01

**Authors:** Hanjin Jeong, Sohee Kim, Manjae Gil, Sanghoon Song, Tae-Ho Kim, Kyung Jin Lee

**Affiliations:** 1 Department of Chemical Engineering and Applied Chemistry, College of Engineering, Chungnam National University, 99 Daehak-ro (st), Yuseong-gu, Daejeon 305-764, Korea; gkswls924@gmail.com (H.J.); manje87@naver.com (M.G.); 2 Center for Membranes, Korea Research Institute of Chemical Technology, 141, Gajeong-ro, Yuseong-gu, Daejeon 34114, Korea; ksoheui@krict.re.kr; 3 Department of Chemical and Biological Engineering, Seoul National University, Seoul 151-742, Korea; 4 Institute of Technology, Ylemtechnology, 419-13, Sandanjungang-ro, Yeosu-si, Jeollanam-do 59613, Korea; songsh5@ylemtech.co.kr

**Keywords:** poly-1-butene, nanofiber, lithium ion battery, shutdown

## Abstract

Nonwoven nanofiber webs from polyolefin show great potential in various fields such as nanofilters, high performance membranes and separators in lithium ion batteries (LiB). Although nonwoven microfiber webs can be obtained by the well-established melt-blown method, it is relatively difficult to produce nonwoven nanofiber web using polyolefin (polyethylene and polypropylene). There have been several reports on the preparation of polyolefin nanofibers by melt-electrospinning, although this approach presents several intrinsic disadvantages, i.e., high processing costs, the requirement of complex equipment, and poor control over pore size or fiber diameter. Solution-based electrospinning has the potential to overcome the drawbacks of melt-electrospinning, but the solubility of most polyolefin is poor. In this study, we found that poly-1-butene, a member of the poly(alpha-olefin) family, can be used in the electrospinning process. We set the concentration of the polymeric solution for electrospinning at 0.65–1.7 g/mL. Here, we report on the fabrication of nonwoven fiber webs composed of poly-1-butene and their copolymers. The diameter of the nonwoven fiber mat was 0.2–0.4 μm, which can be applicable for shutdown layer. As a representative application, we prepared a poly-1-butene nanofiber separator with an appropriate pore size by electrospinning for use as the shut-down layer of a next-generation LiB. The PB-based nanofiber mat provided shutdown ability at around 100 to 120 °C.

## 1. Introduction

At present and in the future, lithium ion batteries (LiBs) are essential devices [1,2,3], but lots of work remains to be done on each component to improve their performance in terms of power, capacity and safety [4]. Notably, the development of novel types of separators is required [5]. The most widely used separators at present are stretched polyethylene (PE) or isotactic polypropylene (PP) with an additional ceramic layer to maintain mechanical and thermal stability [6,7,8,9]. Normally, the pore size of the stretched PE is around 30–100 nm [10,11,12]. Because this is sometimes problematic in the development of high power LiBs, many studies on the modification or replacement of PE have been conducted recently [13,14,15,16,17].

In order to improve the flux of Li ions, nonwoven type separators are being actively investigated [18,19,20]. In general, cellulose or polyimide have been adopted [21,22] as they are electrochemically and thermally stable enough to be used in LiBs.

Although stretched PE separators present several issues for future LiBs, one of their great benefits is improved safety thanks to the shutdown function that they provide [10,23]. When a current leak occurs inside a LiB, the internal temperature will rise, causing fire or an explosion. PE will melt above a certain temperature, leading to the blockage of its nanosized inner pores and preventing further temperature increases. If a stretched PE separator is substituted with different materials [13] or removed in the case of solid-state LiBs, an additional shutdown layer [14,15,16,17] will be favorable to improve the safety of the LiB.

Considering the future requirements of high-power LiBs, nonwoven type shutdown layers will be advantageous [24,25,26]. Nonwoven membranes made of PE are normally fabricated by a melt-blown process [27]. However, the large pores (typically over a few microns in size) that result from this approach make the material unsuitable for application as either the separator and shutdown layer [28,29]. Nonwoven nanofiber mats produced by the electrospinning process [30], for example, are generally accepted as good candidates for next generation separators [31]. Although PE is a perfect material for shutdown layers and separators, it is extremely difficult to obtain nanofiber mats by electrospinning PE or polypropylene (PP) because of the poor solubility of these substances in the solvents typically used for electrospinning [32,33,34].

Poly-1-butene (PB) and derivatives, which are the members of poly(alpha-olefin) family, are perfect candidates in this sense because their electrochemical properties and melt temperature [35,36,37] are very similar to those of PE or PP. In addition, the solubility of the PB family is much better than that of PE or PP, so the production of electrospun nonwoven nanofiber mats is possible [38,39]. Herein, we present the preparation of a nonwoven nanofiber mat from PB homopolymer (hPB) and a PB based copolymer (cPB) as a shutdown layer for a LiB. Various solvent combinations were tested for the electrospinning process. A nonwoven nanofiber mat with a single fiber diameter of 0.2–0.4 μm was obtained. The membrane thickness of the nonwoven nanofiber mat could be controlled by adjusting the spinning time. Finally, the shutdown properties were tested in a metal-separator-metal coin cell structure at various temperatures.

## 2. Materials and Methods

### 2.1. Materials

PE, PP, hPB (poly-1-butene, average *M*w = 750,000) were purchased from Ylem Technology (Yeosu, Korea). cPB (BL series, BL2491M, average *M*w = 470,000) was obtained from MITSUI Chemicals, Inc. (Tokyo, Japan) cPB contains poly-1-butene and polypropylene. Cyclohexane (99.0%), *N*,*N*-dimethylformamide (DMF, 99.5%) and tetrahydrofuran (THF, 99.8%) were purchased from SAMCHUN (Seoul, Korea) and used without further purification.

### 2.2. Preparation of Electrospinning Solution and Polymer Fibers

To prepare for the electrospinning of PB fibers, hPB or cPB were dissolved in cyclohexane at 70 °C, and several cosolvents such as THF and DMF were added. The weight/volume percent (*w*/*v* %) of the polymer and solution was 0.65–1.7 g/mL. The weight ratio of the cosolvent was 1:1:0.1–0.3 of cyclohexane/THF/DMF. The prepared solution was pumped through a needle with metal-tip at a pumping rate of 0.4–0.6 mL/h using a syringe pump (NE-1000, New Era Pump systems, Inc. Farmingdale, NY, USA), and a positive electrical potential of 12–15 kV was applied with a power supply (SHV50R, Conver tech, Seoul, South Korea). The ground electrode was connected to aluminum foil and used as a collector. The distance between the needle and the collector was set to around 14 cm.

### 2.3. Characterization

To analyze functional groups, Fourier transform infrared (FTIR) spectra of each resin were acquired using FTS-175C (Bio-Rad Laboratories, Inc., Cambridge, MA, USA) within the range 4000–700 cm^−1^ by the attenuated total reflection (ATR) method. Differential scanning calorimetry (DSC) (N-650, Sinco Co., Ltd., Seoul, South Korea) was used to determine the melting point. The DSC sample weight was 10 mg and measurements were carried out under a nitrogen atmosphere within a temperature range of 25–300 °C. Scanning electron microscopy (SEM) was conducted with an S-4800 (Hitachi, Tokyo, Japan) with 15 kV of accelerating voltage after platinum (Pt) sputtering (60 s) onto the samples to confirm the morphologies of the fibers.

### 2.4. Preparation of PB Fibrous Separator and Shutdown Test

Under the above spinning conditions, PB nanofibers were deposited onto a commercialized nonwoven fiber support. A poly(ethylene terephthalate) (PET, Mitsubishi) single layer was used as the nonwoven fiber support to prevent the separator from losing mechanical integrity above the temperature at which the PB begins to melt. The prepared nonwoven PB nanofiber mat on PET support was pressurized at about 98 N/cm^2^ and heated at 40 °C for 10 min. A shutdown test to determine the high-voltage breakdown limit of these separators was conducted with a ZIVE MP2 multichannel electrochemical workstation (WonATech, Seoul, Korea). This device was measured using a multifrequency impedance analyzer, while the separator soaked in the electrolyte was maintained under constant pressure at 55 kPa between parallel stainless-steel platelet electrodes [40]. In the experiment, the temperature range was set to 25–200 °C and the heating rate was 2 °C/min

## 3. Result and Discussion

**Figure 1:**
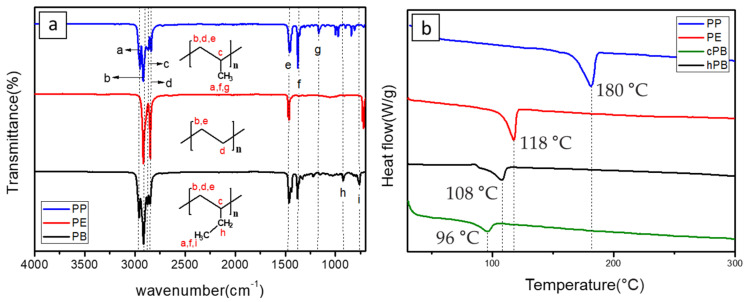
(**a**) FTIR spectra and (**b**) DCS thermograms of PP, PE, cPB, and hPB.

Table 1 summarizes the representative properties of the PE, PP, and PB families, including their molecular structure, and Figure 1a shows the FTIR spectra of PE, PP, and PB. Judging from these FTIR spectra, one can expect the different structures of each polyolefin described in Table 1. The FTIR characteristic peaks of the materials are summarized in the Table 2 [41,42,43,44,45]. Distinct peaks were observed at wavenumbers 2912, 2867, and 1461 cm^−1^ in all cases; [42] 2912 cm^−1^ from the asymmetric stretching of CH_2_, and 2844 cm^−1^ from symmetrical stretching of CH_2_. Compared to PE, PP and PB contained abundant dangling CH_3_ and CH moieties, and FTIR spectra showed their characteristic peaks at 2955 cm^−1^ from the asymmetric stretching of CH_3_, 2867 cm^−1^ from stretching of CH, 1461 cm^−1^ from symmetrical bending of CH_3_, 1370 cm^−1^ from symmetrical bending of CH_3_ and 1163 cm^−1^ from rocking of CH_3_ [41,43,45]. In case of PB, absorption peaks were observed at 921 and 762 cm^−1^, representing rocking of CH_2_ and CH_3_, respectively [43,44].

PB has similar properties to PP and PE, but in terms of solvent solubility and corresponding electrospinnability, it is much better than PP and PE. In addition, cPBs (mainly composed of units of 1-butene and ethylene or propylene) not only have similar solvent properties to PB, but also show melting temperatures which are tunable with the copolymer composition (see Melting Temperature in Table 1). Figure 1b shows a DSC thermogram for each polyolefin used in this study. The melt temperature of polyolefin (especially PE) varied with respect to its molecular configuration, crystallinity and molecular weight; however, it is generally accepted that melting temperatures (*T*_m_) of PP and PE are 180 and 118 °C, respectively. On the other hand, the *T*_m_ of PB is obviously lower than those of PP or PE; this might be because of their reduced crystallinity [46]. Considering that lower shutdown temperatures are sometimes required (especially in sodium ion batteries), PB may be a perfect material for a novel shutdown layer. In addition, as presented in Figure 1b, cPB showed a different melting temperature from hPB. The melting temperature of polymer varies according to the ratio of the polymer’s composition, implying that one can make a shutdown layer with a tunable shutdown temperature. Therefore, hPB and cPB can be used as the shutdown layer in LiBs with tunable shutdown temperatures if nonwoven nanofiber structures are successfully realized using electrospinning.

Although it is almost impossible to find a proper solvent for PE and PP, especially at room temperature, PB provides better solvent properties compared to PE and PP, as mentioned above. Several good solvents for PB are described in the literature [47,48], and through our own feasibility tests, and cyclohexane was shown to be the best to prepare an electrospinnable polymeric solution. There seem to be no reports to date considering the solvent parameters of PB (especially Hansen solubility parameters), but one can expect that *R*_0_ of PB will be greater than that of PE and PP (R_0_ of PE and PP is known to be 2 and 6 MPa^0.5^, respectively) [49,50]. R_0_ is the radius of the interaction, which means the radius of the solubility sphere of a certain polymer in Hansen’s solubility triangle-diagram. These solubility spheres are shown at the boundary point between solubility and insolubility to several solvents in Hansen’s diagram. *R*_a_ means the distance between the solvent and the polymer in Hansen space. These values can be calculated from the solubility parameter components of the solvent and polymer. If *R*_a_ of a certain solvent to polymer is larger than *R*_0_ of the polymer, the solvent will be located in the outer circle of the soluble range in the Hansen space, and as such, that solvent will not be able to dissolve the polymer.

Here, we propose a solubility parameter of PB calculated using the Hoftyzer-Van Krevelen equation for the first time, as presented in Table 3. Each of the Hoftyzer-Van Krevelen solubility parameters can be calculated using the following equations [51]:

(1) $$\delta_{d}=\frac{\sum F_{di}}{V},\;\delta_{p}=\frac{\sqrt{\sum F_{pi}^{2}}}{V},\;\delta_{h}=\sqrt{\frac{\sum E_{hi}}{V}}$$

where *F_di_*,$\;F_{pi}^{2}$, *E_hi_*, and *V* represent the group dispersion component, the group polar component, the hydrogen bonding component and the molar volume, respectively [51]. The solubility parameter components δ*_d_*, δ*_p_* and δ*_h_* indicate dispersion forces, polar interaction and hydrogen bonding, respectively. These parameters are obtained based on the molecular structure itself, but they are comparable with the Hansen solubility parameters as listed in Table 3. In addition, here, we suggest *R*_0_ values of PB based on several solvent behaviors of PB from the literature and from our experiments (Table 4) [52]. Considering the known *R*_0_ value of PE and PP, [49,50] the suggested *R*_0_ value of PB is quite reasonable (7.9–10.7) at room temperature.

All the above results suggest that the selection of a proper solvent for PB will be much easier than for PE or PP. Considering a wide range of good solvents is important in finding a proper solvent for electrospinning, because there are several intrinsic requirements, such as solubility, conductivity, surface tension and volatility, etc. First of all, the concentration of the solution should be higher than the minimum concentration of entanglement formation, where the entanglement of polymer chains is maintained during the electrospinning process. The measurement of entanglement is generally made according to the viscosity of the polymer solution [53]. The following equation determines the viscosity of a polymer in solution:

ln[*η*] = ln *k* + *a* ln *M*_η_ (2)

where [η] is intrinsic viscosity, *k* and *a* are Mark-Houwink parameters and *M*_η_ is the viscosity-average molecular weight [54,55]. These constants depend upon the type of polymer, solvent and temperature. The value of ‘*a*’ is a function of the polymer geometry. These parameters can be determined experimentally by measuring [η] of the polymer having a particular molecular weight. In addition, we have to consider the theta condition of the polymeric solution in each solvent, the molecular weight of polymers, etc. Considering the aforementioned solubility parameters of PB, cyclohexane will be a good candidate with which to prepare a suitable electrospinning solution. On the other hand, a totally nonpolar solvent (such as cyclohexane) is not suitable due to its low conductivity. Therefore, we first selected a good solvent (cyclohexane) to dissolve PB at a high enough concentration, and then added a second solvent to increase the electrospinnability. In this study, we selected THF as the second solvent, and added it to the PB cyclohexane solution according to above volume ratio. No specific precipitations were observed until the ratio of cyclohexane and THF reached 1:1. When the amount of THF exceeded that of cyclohexane, however, some precipitation of PB was observed; this will be a crucial disadvantage for long-term electrospinning. Finally, a certain amount of DMF was introduced to reduce the volatility of the solvent. If the evaporation of the solvent occurs too quickly, solid formation will occur from the jet points, representing another hurdle for long-term jetting [56].

**Figure 2:**
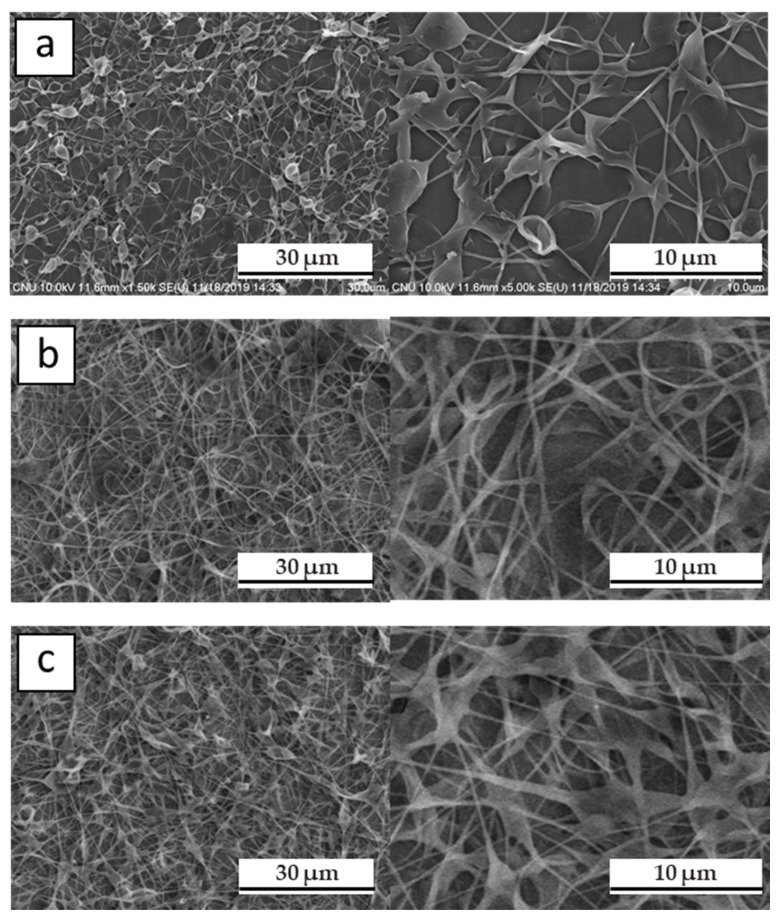
SEM images of electrospun hPB fiber with different volume ratios: Cyclohexane/THF/DMF (**a**) 1:1:0.1, (**b**) 1:1:0.2 and (**c**) 1:1:0.3.

Figure 2 shows SEM images of a hPB nanofiber mat obtained by controlling several conditions; the morphology of the resulting nanofiber was influenced many such conditions, as outlined above. In addition, because these conditions are related to each other, it is extremely difficult to find optimal experimental conditions for electrospinning. However, by observing the fiber morphology under certain conditions, we can find optimized conditions after several trials, as illustrated below (the following will be just one example of an attempt to find optimized conditions for electrospinning). For example, as shown in Figure 2a, some fiber structures were obtained using a 1 *w*/*v* % of PB solution in cyclohexane/THF/DMF (1:1:0.1), but lots of bead on a string structures occurred. It was observed that 1 *w*/*v* % of polymeric solution was too low to yield enough entanglement, but because the molecular weight of hPB is high (750,000 g/mol), these concentrations were fine in this case. The formation of bead on a string structures during electrospinning was the result of competition between the surface tension of the solution and elongation forces from accumulated charge. In this case, because THF is more conductive (1.5 × 10^−6^ S/m) [57] than cyclohexane [58], the charge will be relaxed in solution, resulting in a weak elongation force. In order to increase charge accumulation on the surface, we increased the amount of DMF. Also, the dielectric constant value of DMF was high, which improved the electrospinnability in general [59]. With increasing the amount of DMF, the fibrous structures became more well defined, as shown in Figure 2b,c. In Figure 2b,c, *w*/*v* % was 0.7 and 0.65 and the volume ratio of cyclohexane/THF/DMF was 1:1:0.2 and 1:1:0.3, respectively [46,60].

**Figure 3:**
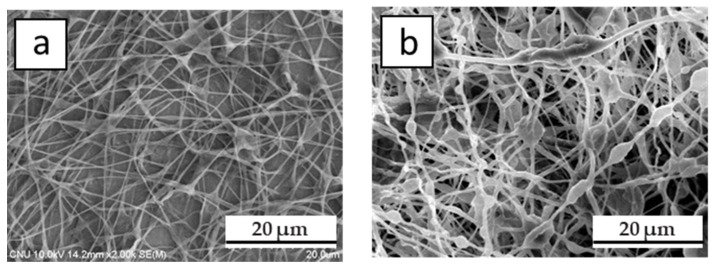
SEM images of electrospun mat under optimized conditions: (**a**) hPB and (**b**) cPB.

Detailed investigation is required to check the degree of chain entanglement with respect to the solvent composition. In addition, the solubility of PB in each solvent (or cosolvent) should be further compared. However, our main objective in this work was to check the shutdown ability using a nonwoven nanofiber composed of PB, and thus, we just tried to find the best conditions to obtain such a material in several trials. Figure 3 shows SEM images of a hPB and cPB nanofiber mat created with optimized conditions. The nanofiber shown in Figure 3a,b was obtained using 0.65 *w*/*v* % and 1.7 *w*/*v* % of hPB and cPB solution, respectively. The solvent ratio of cyclohexane/THF/DMF was 1:1:0.3 (applied voltage: 12–15 kV, flow rate: 0.4–0.6 mL/h, applicable humidity: 40%–50%). Although we cannot say that this condition was optimal, we can say that this solution composition and electrospinning condition were quite robust to obtain a nonwoven nanofiber mat for use as a shutdown layer. The diameters of single nanofibers in the mat were around 0.2–0.4 μm, i.e., much smaller than those in melt-blown fibers. In addition, more importantly, this condition makes it possible to produce electrospun nanofibers using a long enough process time to control the mat thickness by controlling the spinning time. There was no specific breakage of electrospinning under this condition. In our various trials, the growth rate of the membrane thickness was determined as being 15–20 μm/hour.

**Figure 4:**
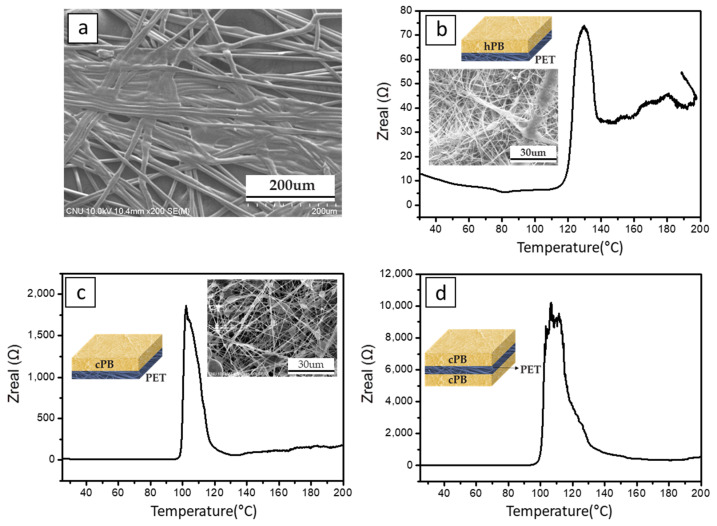
SEM image of (**a**) PET support and Shutdown test data of hPB and cPB fibrous membranes: (**b**) hPB on one side, (**c**) cPB on one side and (**d**) cPB on both sides.

If the separator of the LiB provides a shutdown function at a specific temperature, it will effectively prevent the explosion of the battery [13,61]. Separators containing a PB shutdown layer were prepared by electrospinning PB onto a PET support. Figure 4a shows an SEM image of the PET supporter. The SEM image of the commercial PET supporter (also nonwoven type) shows that the single fiber diameter and pore size are 10 and *ca.* 24 μm (from poro-meter), respectively. The hPB and cPB fibrous membranes were collected onto a PET supporter (inset of each figure). The impedance profiles for the PET separator comprising an electrospun PB nanofiber mat are shown in Figure 4b–d. In the inset of each graph, the cell architecture is schematically described; the yellow layer indicates electrospun PB and the blue indicates the commercial PET support. Representative SEM images of PB nanofibers on a PET supporter are also provided in Figure 4b,c; these images show the successful electrospinning procedure, even onto a PET supporter. In Figure 4b, shutdown was observed starting at 120 °C; at this temperature, the cell impedance increased sharply. This starting temperature is well-matched with the melting point of hPB. In the case of cPB, the level of regularity and crystallinity were reduced, which led to a lower melting point [62]. Accordingly, it was observed that cPB showed a lower shutdown temperature than hPB. Figure 4c,d show that the starting shutdown temperature was around 100 °C, reflecting the melting temperature of cPB. When the shutdown layer was loaded on both sides of the PET separator, the impedance of the separator (Figure 4d) was much higher than that with a shutdown layer on just one side (Figure 4c; here, the total amount of cPB was same in both cases). The impedance graph as a function of temperature shows the typical shutdown behavior of commercial PE separators, and reveals the effect of the successful introduction of a nonwoven type PB shutdown layer.

## 4. Conclusions

In conclusion, we have successfully prepared an electrospun PB nanofiber mat that is expected to be applicable as the shutdown layer on a LiB separator. Using a cosolvent of cyclohexane/THF/DMF, hPB or cPB nonwoven nanofiber mats can be produced by optimizing the electrospinning procedure. Furthermore, a separator with a tailored shutdown function was successfully manufactured by electrospinning hPB or cPB on a PET support. During testing, it was found that the shutdown started at a temperature similar to the melting point found by DSC in all cases. In addition, it was shown that the separator with an electrospun cPB on both sides had a higher resistance value than that with the cPB one just side. This proposed PB-based electrospun nonwoven nanofiber mat could be used as the shutdown layer for future LiBs without a stretched PE separator. These PB-based olefin separators could therefore improve the safety of next-generation LiBs with high-power and large capacities. In addition, these separators will make it possible to produce shutdown layers at the required melting point because the melting point of cPB can be controlled by adjusting the ratio of monomer in the copolymer.

## Figures and Tables

**Table 1:** Summary of several properties of PE, PP and PB.

	PE	PP	PB		
			hPB	cPB (With PP)	
**Structure**	** 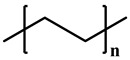 **	** 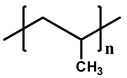 **	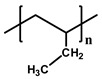	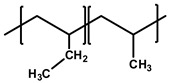	
**T_m_ (°C)**	118	180	108	96	
**Solubility**	N.A for any electrospinning solvent	N.A for any electrospinning solvent	Volume ratio of cosolvent (Cyclohexane/THF/DMF)		
			1:1:0.1	1:1:0.2	1:1:0.3
			soluble	soluble	soluble
**Spinnability**	N.A.*	N.A.	Poor	Good	Best

* N.A.: Not available.

**Table 2:** IR spectra data of PP, PE and PB.

Materials	Wavenumber(cm^−1^)	Vibration Type	Assignment
PP, PB	a (2955)	Asymmetrical stretching	CH_3_
PP, PE, PB	b (2912)	Asymmetrical stretching	CH_2_
PP, PB	c (2867)	stretching	CH
	d (2844)	Symmetrical stretching	CH_2_
PP, PE, PB	e (1461)	Symmetrical bending	CH_2_
PP, PB	f (1370)	Symmetrical bending	CH_3_
PP	g (1163)	Rocking	CH_3_
PB	h (921)	Rocking	CH_2_
	i (762)	Rocking	CH_3_

**Table 3:** Solubility parameters of Hansen and Hoftyzer-Van Krevelen group contribution methods.

Solubility Parameter	Hansen		Hoftyzer-Van Krevelen		
	PE ^a^	PP ^a^	PE ^b^	PP ^b^	PB ^b^
δ_d_	18	18	16.4	19.7	18.9
δ_p_	0	0	0	0	0
δ_h_	2	1	0	0	0
*R*_0_	2	6			

^a.^ Ref [49,50]. ^b.^ Calculated using the Hoftyzer-Van Krevelen group contribution method [51].

**Table 4:** Hansen solubility parameter and solvent behaviors of PB in common organic solvents. Unit: MPa^0.5^.

Solvent	δ*_d_*	δ*_p_*	δ*_h_*	*R_a_*	Solvent Behavior
Methanol	14.7	12.3	22.3	26.8	insoluble
DMF	17.4	13.7	11.3	18.0	insoluble
Acetone	15.5	10.4	7	14.2	insoluble
Methyl Acetate	15.5	7.2	7.6	12.4	insoluble
THF	16.8	5.7	8	10.7	insoluble
Hexane	14.9	0	0	7.9	soluble
Cyclohexane	16.8	0	0.2	4.1	soluble
Benzene	18.4	0	2	2.2	soluble
